# CHK1 and RAD51 activation after DNA damage is regulated via urokinase receptor/TLR4 signaling

**DOI:** 10.1038/cddis.2016.291

**Published:** 2016-09-29

**Authors:** Pavan B Narayanaswamy, Sergey Tkachuk, Hermann Haller, Inna Dumler, Yulia Kiyan

**Affiliations:** 1 Department of Nephrology, Hannover Medical School, Hannover D-30625, Germany

## Abstract

Mechanisms of DNA damage and repair signaling are not completely understood that hinder the efficiency of cancer therapy. Urokinase-type plasminogen activator receptor (PLAUR) is highly expressed in most solid cancers and serves as a marker of poor prognosis. We show that PLAUR actively promotes DNA repair in cancer cells. On the contrary, downregulation of PLAUR expression results in delayed DNA repair. We found PLAUR to be essential for activation of Checkpoint kinase 1 (CHK1); maintenance of cell cycle arrest after DNA damage in a TP53-dependent manner; expression, nuclear import and recruitment to DNA-damage foci of RAD51 recombinase, the principal protein involved in the homologous recombination repair pathway. Underlying mechanism implies auto-/paracrine signaling of PLAUR/TLR4 receptor complex leading to activation of CHK1 and DNA repair. The signaling is induced by a danger molecule released by DNA-damaged cells and mediates, at least partially, activation of DNA-damage response. This study describes a new mechanism of DNA repair activation initiated by auto-/paracrine signaling of membrane receptors PLAUR/TLR4. It adds to the understanding of role of PLAUR in cancer and provides a rationale for therapeutic targeting of PLAUR/TLR4 interaction in TP53-positive cancers.

Therapeutic efficiency of many cancer chemotherapeutic drugs and radiotherapy depends on the induction of DNA damage. DNA damage can vary from single-strand breaks to double-strand breaks (DSBs) to complex chemical modifications of bases. Accordingly, the cells have evolved numerous intricate repair mechanisms for specific kinds of damage. DSBs are the most lethal, as they can lead to chromosomal aberrations and translocations. Two major pathways to deal with DSBs are homologous recombination repair pathway (HR) and non-homologous end joining (NHEJ). Generally, detection of DNA damage leads to cell cycle arrest, regulation of DNA replication and activation of the repair pathway. Ability of a cell to repair or bypass DNA damage determines the choice of cell fate leading to cell survival, senescence or apoptosis.^1^

Detection of DNA lesions by so-called DNA-damage sensors leads to activation of apical ATM (ataxia telangiectasia mutated) and ATR (ATM and Rad3-related) kinases and their recruitment to the DNA-damage sites. Checkpoint kinase 1 (CHK1) is one of the key downstream molecules of DNA-damage response (DDR) signaling. In response to DNA damage, CHK1 is phosphorylated at Ser345 primarily by ATR kinase,^2^ to arrest the cell cycle in S and at G2/M phases that promote DNA repair before cell division. Multiple further functions of CHK1 in regulation of transcription and cell metabolism are just emerging.^3, 4^ It was also reported, that CHK1 can be phosphorylated by other kinases (PKB/AKT and MAPKAPK, p90/RSK) at different sites.^4^ Though this phosphorylation affects functions and intracellular distribution of CHK1, clear understanding of CHK1 regulation is still missing. CHK1 phosphorylates a variety of intracellular substrate proteins including the recombinase RAD51, the central molecule in HR pathway that binds single-strand DNA at the sites of damage-forming filaments that are observed microscopically as nuclear foci. RAD51 filament formation is essential for homology search and strand exchange. RAD51 overexpression is observed in many cancers and is associated with an increased efficiency of DNA repair and resistance to chemotherapy.^5^

DDR is not limited to nuclear activation of DNA repair machinery. Communication between irradiated and unirradiated bystander cells results in DNA-damage induction in the latter as a result of so-called bystander effect (BE).^6^ It is believed that this communication is mediated by direct cell–cell contacts or release of soluble factors. Furthermore, damaged cells benefit from the feedback rescue signal of bystander counterparts.^7^ BE has important therapeutic significance because it can compromise efficiency of irradiation and cause deleterious effects in off-target healthy tissues. Several soluble factors have been suggested to be mediators of BE.^6^ However, detailed understanding of BE and rescue signaling are still missing.

Urokinase plasminogen activator receptor (PLAUR) is a GPI-anchored receptor, which binds its ligand, a serine protease urokinase-type plasminogen activator (PLAU). PLAU/plasmin–activated proteolytic cascades promote cell invasion through remodeling of the extracellular matrix. PLAUR does not possess any intracellular or transmembrane domains, however, it can induce intracellular signaling via interaction with other receptors.^8^ Expression of PLAUR in quiescent tissues is low, whereas its overexpression has been observed in many cancers and is associated with poor survival and prognosis.^9^ Over the last decades significant amount of experimental data provided evidence for multiple roles of PLAUR in cancer biology (reviewed recently in ref. 9). These data justify the attempt to use PLAUR as a target for cancer therapy. However, inhibition of proteolytic function of PLAUR was not efficient in clinical trials,^10^ strengthening the importance of proteolysis-independent functions of PLAUR in cancer.

Our recent findings revealed a link between PLAUR and DNA damage-induced activation of the ubiquitin-proteasome system,^11^ resulting in delayed DNA repair.

In the present work, we address the mechanisms linking PLAUR to the DNA repair machinery. We show that PLAUR/TLR4 signaling mediates, at least partially, activation of CHK1 and its downstream target RAD51 as a part of auto-/paracrine signaling loop, resulting in more-efficient DNA repair. Accordingly, silencing PLAUR expression results in delayed DNA repair and decreased cell survival in a TP53-dependent manner. This auto-/paracrine pathway is initiated by a molecule released from the nucleus of damaged cells and complements a response initiated in the cell nucleus by DNA-damage sensor proteins. Our data open a new perspective for therapy by obstructing DNA repair processes via targeting membrane receptors.

## Results

### PLAUR increases the efficiency of DNA repair

**Figure 1:**
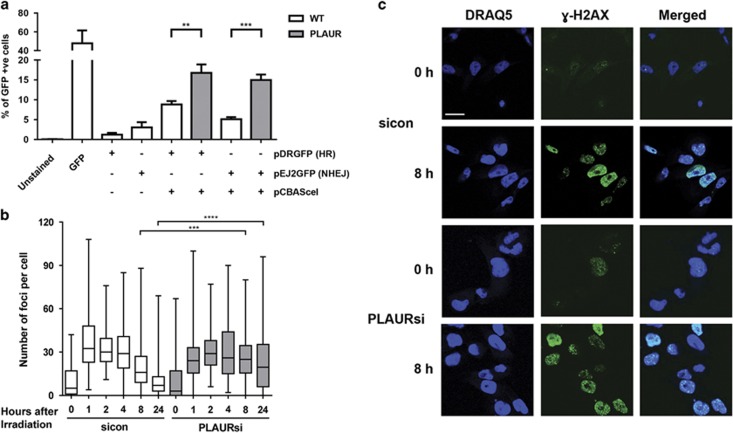
PLAUR expression promotes DNA repair. (**a**) DNA repair assay using plasmid substrates pDRGFP (for HR) and pEJ2GFP (for NHEJ), in WT and PLAUR-expressing HEK-293 cells. Cells were transfected with pCBASceI for the introduction of a double-strand break and DNA repair efficiency correlating with GFP expression was assessed after 3 days by FACS. Data shown as mean±S.D. (**b**) MDA-MB-231 were irradiated at 5 Gy and fixed after indicated time points, kinetics of *γ*-H2AX foci formation was then studied by performing immunofluorescence. The number of foci per cell was quantified using ImageJ. Data shown as mean ±S.D. (**c**) Representative images of *γ*-H2AX foci. Scale bar 20 μm

Our earlier observations using comet assay have shown that DNA repair was delayed in PLAURsi vascular and MDA-MB-231 cancer cells.^11^ To gain more direct insight into the mechanisms regulated by PLAUR, we performed *in vitro* DNA repair assay using reporter plasmid substrates.^12^ We used HEK-293 cells that do not express endogenous PLAUR. Control and PLAUR-transfected cells were transfected with pCBASceI and either pDRGFP (for HR) or pEJ2GFP (for NHEJ). GFP expression was assessed by FACS, which showed that PLAUR-expressing cells exhibited increased DNA repair efficiency when compared with wild-type (WT) cells and also that PLAUR can influence both the HR and NHEJ repair pathways (Figure 1a).

Phosphorylation of H2AX and formation of nuclear foci serves as an indicator of DNA damage.^13^ To assess the efficiency of endogenous DNA repair we next irradiated PLAURsi MDA-MB-231 cells and stained for *γ*-H2AX. The number of *γ*-H2AX foci per cells was quantified and showed that in control cells the number of foci increased after irradiation and decreased after 8 h, indicating efficient repair of DNA-damage lesions and foci resolution. On the contrary, in PLAURsi cells a significant number of foci persisted even after 24 h–indicating that DNA repair was impaired (Figures 1b and c).

### PLAUR affects RAD51 expression, phosphorylation and its nuclear translocation

**Figure 2:**
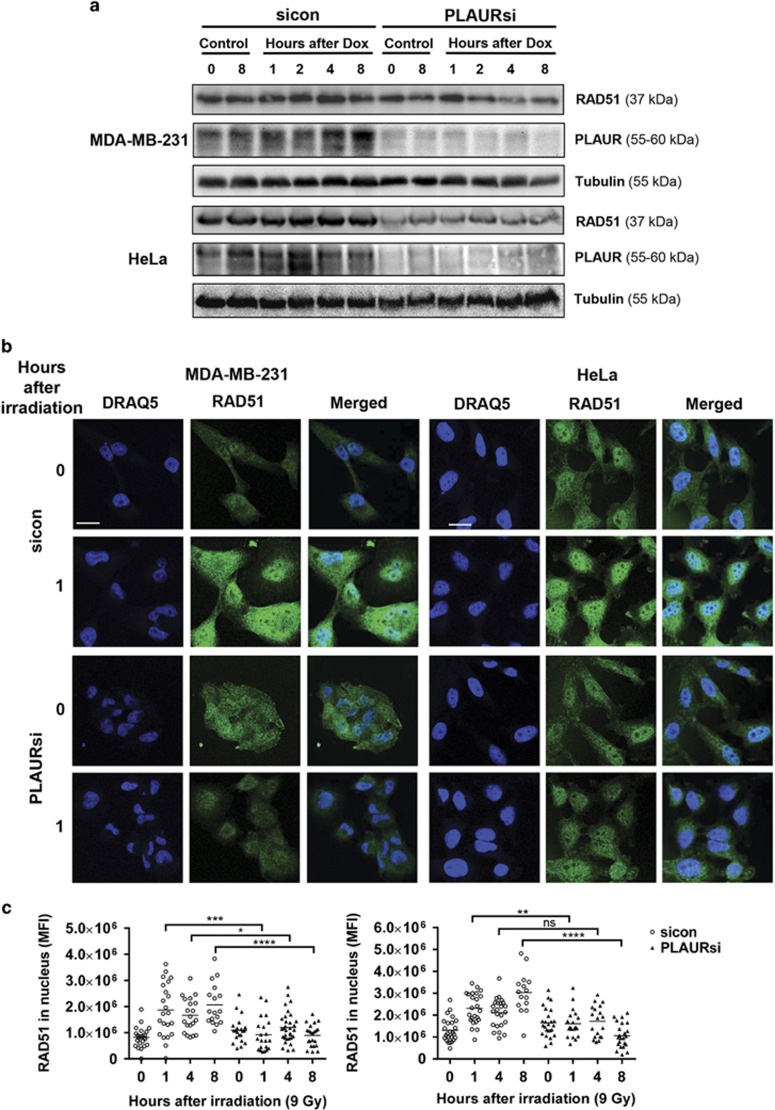
Expression and nuclear import of RAD51 is impaired in PLAURsi cells. (**a**) sicon and PLAURsi, MDA-MB-231 and HeLa cells were synchronized by double thymidine block and treated with Dox (2 *μ*M) for 1 h, and lysates were made at indicated time points. Western blotting was performed to check for the expression of RAD51. (**b**) MDA-MB-231 and HeLa cells silenced for PLAUR were irradiated at 9 Gy, fixed after indicated time points, and nuclear translocation and foci formation of RAD51 was detected by immunofluorescence. Scale bar 20 *μ*m. (**c**) Graphs showing mean fluorescence intensity (MFI) of RAD51 in the nucleus

As PLAUR was affecting both the DNA DSB repair pathways, we looked at expression of some important proteins mediating these pathways. MDA-MB-231 and HeLa cells were synchronized using double thymidine block, released from the block and treated with Doxorubicin (Dox) for 1 h, followed by western blotting. We observed that in both the cell lines, silencing control (sicon) cells showed an upregulation of RAD51 protein expression upon DNA damage, whereas in PLAURsi cells RAD51 levels were downregulated (Figure 2a). Similar results were obtained in MDA-MB-231 after irradiation (Supplementary Figure S1A).

As we observed that the NHEJ pathway can also be influenced by PLAUR (Figure 1a); we analyzed the expression of KU80, the primary protein involved in the NHEJ pathway and observed that levels of KU80 increased on DNA damage in sicon cells, but in PLAURsi cells basal levels of this protein were already high (Supplementary Figure S1A). The significance of PLAUR in the regulation of NHEJ has to be investigated further.

On DNA damage, RAD51 undergoes nuclear translocation and forms filaments at damaged sites that can be detected microscopically as nuclear foci.^14^ We performed immunofluorescence to detect RAD51 foci formation and observed that in sicon cells, RAD51 expression was upregulated and it accumulated inside the nucleus 1 h after irradiation (Figures 2b and c); after 4–8 h, RAD51 foci are distinctively visible in the nucleus (Supplementary Figure S1B, S2A). Whereas in PLAURsi cells both, upregulation of RAD51 expression and foci formation were impaired.

**Figure 3:**
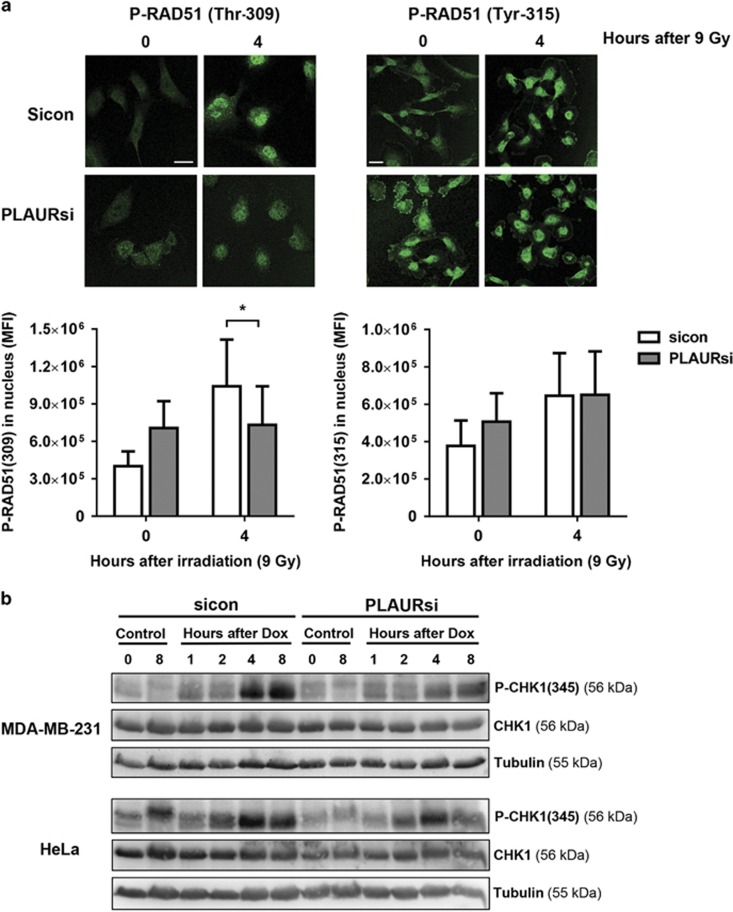
Phosphorylation of RAD51 in PLAURsi cells is abrogated as a result of impaired checkpoint activation. (**a**) sicon and PLAURsi MDA-MB-231 cells were irradiated at 9 Gy and fixed after 4 h. Phosphorylation of RAD51 at Thr309 and Tyr315 was evaluated by immunofluorescence, graphs showing the MFI of P-RAD51 in the nucleus (below). Scale bar 20 *μ*m. Data shown as mean±S.D. (**b**) MDA-MB-231 and HeLa cells silenced for PLAUR were synchronized by double thymidine block and treated with Dox (2 *μ*M) for 1 h, lysates were made at indicated time points. Western blotting was performed to check for the phosphorylation of CHK1 at Ser345

RAD51 has a number of different phosphorylation sites;^15^ it is phosphorylated at Tyrosine-315 by C-ABL, and this increased the nuclear translocation and foci formation ability of RAD51 in chronic myeloid leukemia cells.^16^ CHK1 phosphorylates RAD51 at Threonine-309, and this phosphorylation is necessary for HR repair; as expression of a phosphorylation-deficient mutant or inhibition of CHK1 sensitized the cells to DNA damage.^17, 18^ Having seen a decrease in foci formation in PLAURsi cells, we studied if impaired phosphorylation of RAD51 was the reason behind this observation. We performed immunofluorescence on irradiated MDA-MB-231 cells and found that in sicon cells phosphorylation of both Thr309 and Tyr315 increased after irradiation; whereas in PLAURsi cells Thr309 phosphorylation levels were low and Tyr315 remained unchanged (Figure 3a). We also observed that basal levels of these phosphorylations in PLAURsi cells were high.

### CHK1 activation is impaired in PLAURsi cells

Decreased phosphorylation of RAD51 at Thr309 prompted us to investigate if there was proper activation and functioning of CHK1. Cells were synchronized using double thymidine block and then DNA damage was induced by Dox, cells were analyzed at different time points by western blotting. We found that in sicon cells, expression of CHK1 and its phosphorylation at Ser345 increased significantly at 4 and 8 h after DNA damage, whereas in PLAURsi cells this activation was impaired, much more drastically in MDA-MB-231 than HeLa (Figure 3b). Similar results were obtained after irradiation of these cells (Supplementary Figure S2B).

### PLAUR -TLR4 interaction is involved in the activation of DDR

**Figure 4:**
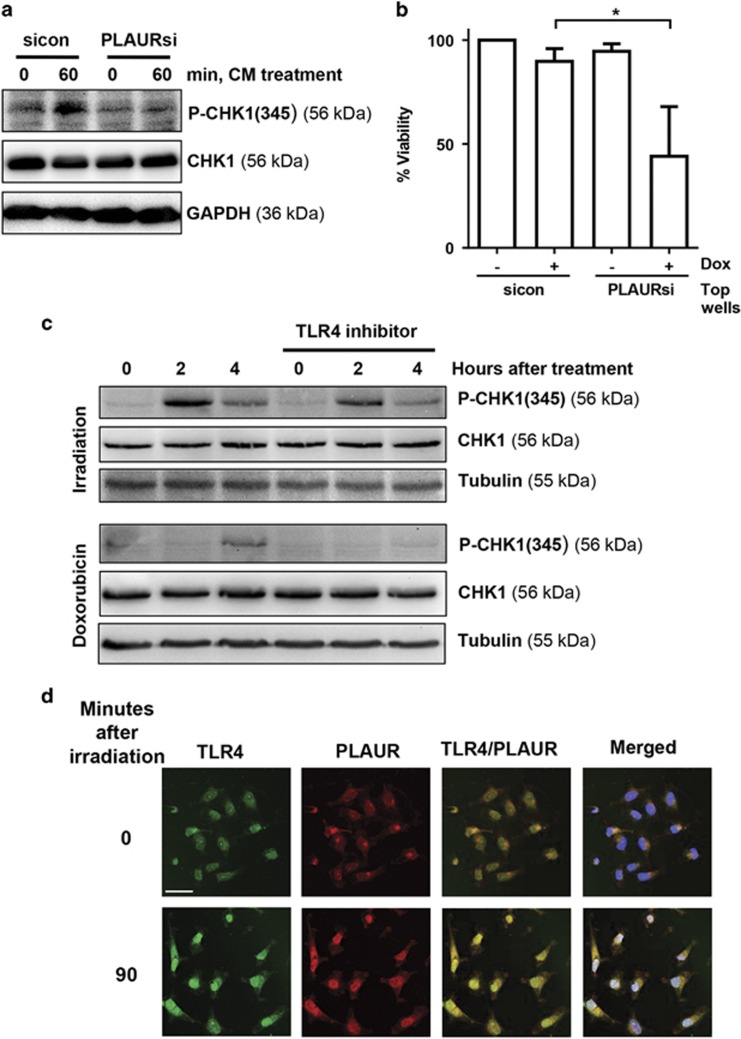
PLAUR/TLR4 signaling is important during DNA damage. (**a**) Normal HeLa cells were treated with 2 *μ*M Dox for 1 h, washed and conditioned media (CM) was collected after 3 h. This CM was added to sicon and PLAURsi HeLa cells and incubated for 60 min, western blotting was performed to determine the phosphorylation of CHK1. (**b**) sicon and PLAURsi HeLa cells in the inserts, were treated with Dox (2 *μ*M) for 1 h, and then placed in co-culture with untreated cells. Viability of the cells in the inserts was assessed after 6 days using CCK-8. Data shown as mean ±S.D. (**c**) HeLa cells pre-treated with TLR4 inhibitor (10 *μ*g/ml) were stimulated with radiation (9 Gy) or Dox (2 *μ*M), lysates were made after 2 and 4 h. Western blotting was performed for phosphorylation of CHK1. (**d**) HeLa cells irradiated at 9 Gy were fixed after 90 min and immunofluorescence was performed to detect colocalization of PLAUR and TLR4. Scale bar 40 *μ*m

It was very intriguing to think about how a cell surface receptor like PLAUR was involved in regulation of an intracellular event like DNA repair. We hypothesized that PLAUR may participate in BE^7^ signaling induced by a soluble factor. In order to prove this, we treated HeLa cells with Dox for 1 h and collected conditioned media after 3 h; this was then applied to undamaged cells and induced CHK1 phosphorylation in sicon but not in PLAURsi cells (Figure 4a).

To investigate the functional consequence of this signaling, transwell co-culture experiments were performed. sicon and PLAURsi cells grown in transwells were treated with Dox, washed with PBS and placed in co-culture with untreated cells. After 6 days viability of the cells was determined using CCK-8. Viability of bystander cells was not affected by co-culture with Dox-treated cells (Supplementary Figure S3A). However, co-culture with undamaged bystander cells improved resistance of sicon cells to the otherwise lethal Dox (Figure 4b). On the contrary, PLAURsi cells failed to sense paracrine rescue signals released by bystander cells to promote DNA repair and showed decreased viability.

As PLAUR does not have any transmembrane domains to induce intracellular events, we looked for its interaction partner. Our recent data showed that PLAUR co-operates with TLR4 to mediate oxidized low density lipoprotein-induced events in vascular cells.^19^ TLR family of receptors mediates innate immunity response to a wide variety of pathogens by recognition of pathogen associated molecular motifs. In addition, TLRs sense so-called damage-associated molecular patterns (DAMP), such as extracellular high mobility group box 1 protein (HMGB1), histones and hemoglobin.^20, 21^ It has been shown that the entire TLR family of proteins and particularly TLR4 is induced by radiation in lymphocytes isolated from healthy subjects.^22^ In addition, deficiency of TLR4 promotes cancer development through decreased expression of DNA repair proteins leading to impaired DNA repair.^23^ HeLa cells pre-treated with vehicle or TLR4 inhibitor were irradiated at 9 Gy and cell lysates were made after 2 and 4 h. Cell pretreatment with TLR4 inhibitor significantly decreased radiation-induced phosphorylation of CHK1; this effect was much more pronounced when DNA damage was induced by Dox (Figure 4c).

**Figure 5:**
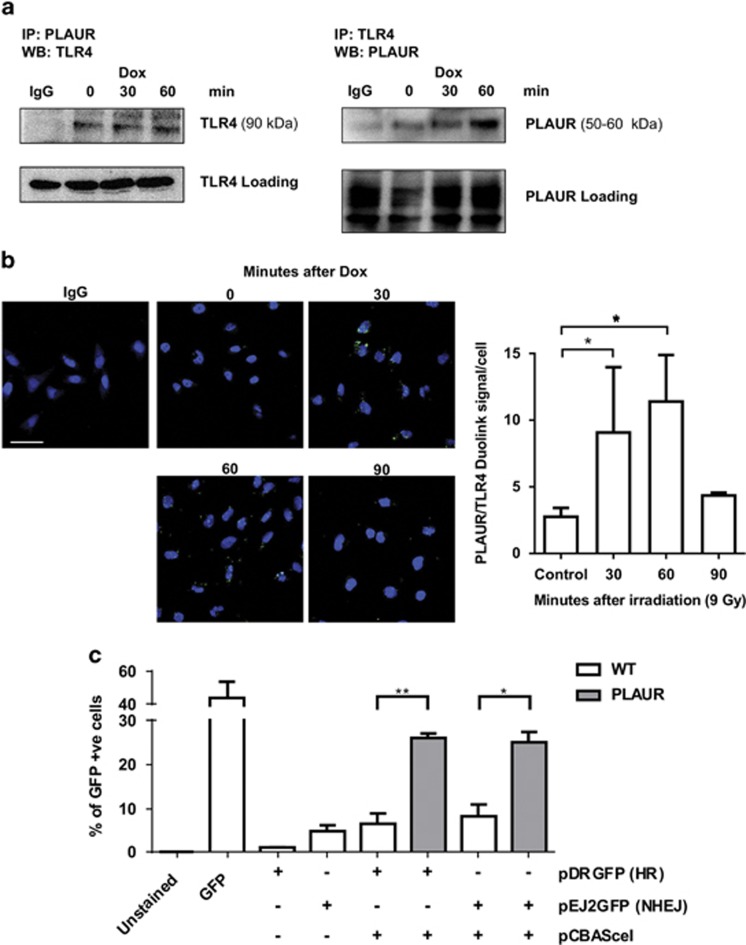
PLAUR/TLR4 interaction increases DNA repair efficiency. (**a**) PLAUR was immunoprecipitated from HeLa cell lysates after Dox treatment, TLR4 was detected in the immunoprecipitates by western blotting. Normalization was performed by the amount of TLR4 present in the original lysates. The experiment was repeated in the reverse direction (Right). (**b**) HeLa cells were treated with radiation of 9 Gy and fixed at the indicated time points, Duolink proximity ligation assay was performed to determine the interaction between PLAUR and TLR4. Duolink signal per cell was counted and represented as a graph (right). Scale bar 20 *μ*m. Data shown as mean ±S.D. (**c**) DNA repair assay using plasmid substrates pDRGFP (for HR) and pEJ2GFP (for NHEJ), in WT and PLAUR-expressing HEK-Blue hTLR4cells having constitutive expression of TLR4, CD14 and MD-2. Cells were transfected with pCBASceI for the introduction of a double-strand break and DNA repair efficiency correlating with GFP expression was assessed after 3 days by FACS. Data shown as mean ±S.D.

We speculated that PLAUR/TLR4 can also induce signaling in response to a danger molecule released by the cells in response to irradiation. We performed immunocytochemical study to analyze whether PLAUR associates with TLR4 after DNA-damage induction. As shown in Figure 4d and Supplementary Figure S3B, irradiation causes colocalization of these receptors. Consistent results confirming interaction of receptors were obtained by co-immunoprecipitation studies (Figure 5a). To analyze whether other members of TLR family can be involved in interaction with PLAUR, its association with TLR1, TLR3 and TLR5 have been studied by co-immunoprecipitation (Supplementary Figure S4A). These and TLR2 are the extracellular receptors of the family. However, HeLa cells do not express any detectable level of TLR2. We detected some interaction of PLAUR with TLR1 which was however decreased after induction of DNA damage. We conclude from this data that it is PLAUR/TLR4 complex, which is involved in BE signaling.

Direct association of PLAUR and TLR4 was further confirmed using Duolink proximity ligation assay on irradiated HeLa cells. Interaction of TLR4 and PLAUR was weakly seen in untreated cells, but increased significantly 30–60 min after irradiation, suggesting direct interaction of the two receptors (Figure 5b).

To further explore the role of PLAUR/TLR4 signaling, DNA repair assay was performed in PLAUR-transfected HEK-Blue hTLR4cells. These engineered cells stably express human TLR4 and its co-receptors MD-2 and CD14. As shown in Figure 5c, HEK-Blue hTLR cells expressing both, TLR4 and PLAUR demonstrate significantly higher efficiency of HR DNA repair pathway compared with PLAUR-expressing HEK-293 cells (3.9-fold *versus* 1.9-fold, Figure 1a).

Nature of extracellular ligand inducing PLAUR/TLR4 response is of interest. PLAU is a natural ligand of PLAUR. We studied PLAU expression and observed its transient increase 4 h after DNA-damage induction (Supplementary Figure S4B). To investigate whether PLAU can induce the observed effects, cells were pre-treated with antibody blocking PLAU/PLAUR binding. As shown in Supplementary Figure S4C this had no effect on CHK1 activation, whereas ERK phosphorylation was decreased. Interestingly, we observed that blocking nuclear export with Leptomycin-B abrogates activation and nuclear import of RAD51 (Supplementary Figure S4D), thus suggesting nuclear origin of the bystander signaling mediator ligand. One further candidate is HMGB1 protein that can be released by cancer cells after irradiation.^24^ However, anti-HMGB1-blocking antibody did not affect Dox-induced activation of CHK1 (Supplementary Figure S5A).

The above data strongly suggest that PLAUR/TLR4 mediate auto-/paracrine signaling from DNA-damaged cells to facilitate CHK1 activation and DNA repair. The nature of the ligand recognized by the receptor complex remains, however, to be determined.

### Functional effects of PLAUR/TLR4 signaling

**Figure 6:**
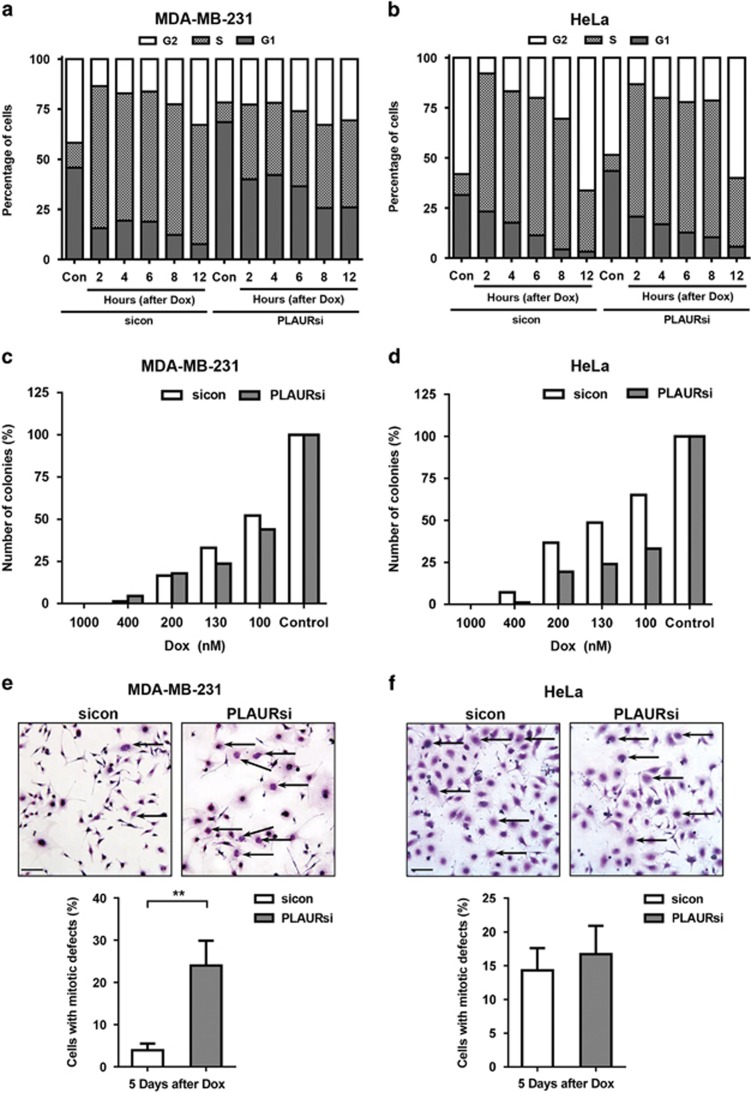
Cell cycle arrest on DNA damage in PLAURsi cells is dependent on TP53 status. (**a** and **b**) Cell cycle analysis of MDA-MB-231 (**a**) and HeLa (**b**), synchronized by double thymidine block and treated with Dox (2 *μ*M) for 1 h, cells were fixed at the indicated time points, stained with propidium iodide and at least 30 000 cells were analyzed by FACS. Graphs represent the percentage of cells in different phases of the cell cycle. (**c** and **d**) sicon and PLAURsi MDA-MB-231 (**c**) and HeLa (**d**) were treated with the indicated concentrations of Dox for 1 h, then trypsinised and plated at low density in 10 cm dishes, colonies formed after 12 days were counted using ImageJ. (**e** and **f**) sicon and PLAURsi MDA-MB-231 (**e**) and HeLa (**f**) were plated at low density and treated with low concentration of Dox (125 nM) for 1 h, washed and incubated for 3 days followed by modified Giemsa staining. Enlarged cells having nuclear mitotic defects are marked with arrows. Graphs depict the population of cells with mitotic abnormalities (below). Data shown as mean ±S.D.

CHK1 activation is necessary for HR and it controls the Intra-S and G2 checkpoints.^17, 18^ Thus, we addressed the impact of impaired CHK1 activation by performing cell cycle analysis on synchronized cells treated with Dox. We found that in response to DNA damage, sicon cells were arrested in the S-phase so as to repair the DNA. Whereas PLAURsi MDA-MB-231 cells failed to activate this checkpoint, and proceeded normally through the cell cycle (Figure 6a; Supplementary Figure S5B). On the contrary, PLAURsi HeLa cells did not seem to inactivate this checkpoint and remained arrested in S-phase (Figure 6b).

Colony-forming assay (Figures 6c and d) further confirmed that PLAURsi HeLa cells have significantly decreased cell survival after DNA damage. The number of colonies in MDA-MB-231 PLAURsi cells was not decreased significantly. We performed modified Giemsa staining for cell morphology and observed that PLAURsi MDA-MB-231 (but not HeLa) showed significantly increased number of enlarged multinucleated cells in comparison to sicon cells,^25^ indicating mitotic defects (Figures 6e and f). Thus, our observations suggested that both, PLAURsi MDA-MB-231 cells and HeLa cells (ref. 11 and Supplementary Figure S5C) have delayed DNA repair; however they differ in their ability to maintain cell cycle arrest.

**Figure 7:**
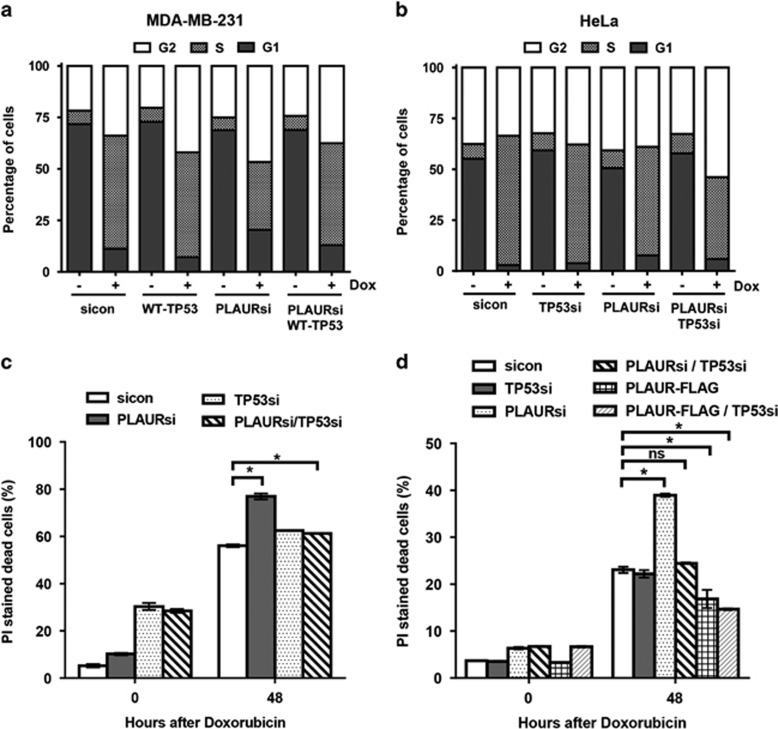
PLAURsi affects the viability of the cells on DNA damage, only in presence of WT-TP53. (**a** and **b**) sicon and PLAURsi MDA-MB-231 cells expressing WT-TP53 and HeLa silenced for TP53, were synchronized by double thymidine block and treated with Dox (2 *μ*M) for 1 h, cells were fixed 8 h after treatment. Cells were stained with propidium iodide and cycle analysis was performed, percentage of cells in the different phases were plotted. MDA-MB-231 (PLAURsi S-phase 32%, PLAURsi+WT-TP53 S-phase 49%); HeLa (PLAURsi S-phase 53.3%, PLAURsi+TP53si S-phase 40.1%). (**c**) PI staining was performed in sicon and PLAURsi HeLa cells 48 h after Dox treatment. Data shown as mean ±S.D. (**d**) sicon and PLAURsi and PLAUR overexpressing MCF-7 cells were transduced with lentiviral constructs to silence (WT) TP53, and treated overnight with Dox (2 *μ*M), after 48 h cells were stained with propidium iodide (PI) and acquired by FACS to detect the number of dead cells. Data shown as mean ±S.D.

We assumed that different responses of the two cell lines could originate from different TP53 status. MDA-MB-231 has mutant TP53 with a missense mutation of Arginine-280 to Lysine, whereas HeLa has WT-TP53.^26, 27^ The roles of CHK1 and TP53 in regulation of intra-S checkpoint has been well documented.^28^ Cyclin-dependent kinases (CDKs) regulate the progression of the cell cycle, and cell cycle arrest can occur when they are inactivated by phosphorylation. CDC25A is a dual specificity phosphatase, which removes the inhibitory phosphorylation of CDKs and allows normal cell cycle progression.^29^ During DDR, CHK1 mediates checkpoint activation by degradation of CDC25A and TP53 controls checkpoint activation by regulating the expression of CDC25A.^28^ To confirm that the checkpoint arrest observed in PLAURsi HeLa cells was owing to the presence of WT-TP53, we expressed WT-TP53 in MDA-MB-231 cells (Supplementary Figure S6A. We observed that sicon and WT-TP53 cells were arrested at S-phase; and PLAURsi cells had significantly lower number of cells arrested in S-phase. However, when WT-TP53 was expressed in PLAURsi MDA-MB-231 cells, the S-phase checkpoint was restored (Figure 7a). Similarly, silencing both WT-TP53 and PLAUR was necessary in HeLa cells to abrogate checkpoint arrest (Figure 7b,Supplementary Figure S6B).

To further verify fate of HeLa cells, propidium iodide staining of dead cells was performed 48 h after Dox treatment. As expected, we did not observe significant cell death in MDA-MB-231 (Supplementary Figure S6C,D). However, increased cell death of PLAURsi HeLa was abrogated after silencing of TP53 (Figure 7c,Supplementary Figure S6E). As TP53 function in HeLA cells can be affected by the action of HPV E6, we verified our data using MCF-7 cells expressing WT-TP53. Both, silencing and overexpression of PLAUR have been performed. Similar to HeLa cells, PLAURsi results in high cell death after Dox treatment (Figure 7d), which is abrogated by TP53si, whereas overexpression of PLAUR decreased cell death. We discern that both TP53 and CHK1 work together to activate the S-phase checkpoint on DDR, but when cells lack functional TP53, the task of activating the checkpoint is influenced by PLAUR through CHK1.

## Discussion

**Figure 8:**
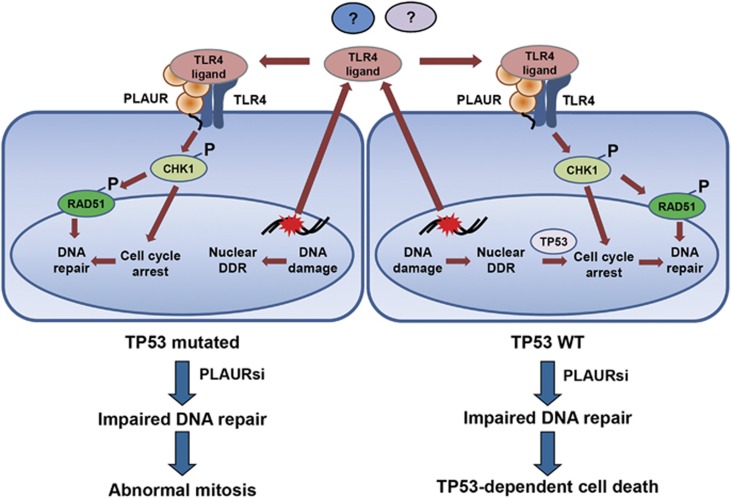
Proposed mechanism of activation of DDR through interaction of PLAUR and TLR4. Damage molecule serving as a ligand of PLAUR/TLR4 receptor complex is released from the cell nucleus. PLAUR/TLR4 auto-/paracrine signaling promotes CHK1 phosphorylation, activation and nuclear import of RAD51. Downregulation of PLAUR in TP53 WT cells results in delayed DNA repair and TP53-dependent cell cycle arrest and cell death. Cell cycle arrest in TP53-mutated PLAURsi cells is compromised and results in abnormal mitosis

Cells have evolved a variety of mechanisms to maintain their genomic integrity. Traditional view on DDR signaling implies that initiation of DNA repair is a chain of nuclear events directed at the activation of repair machinery. Our data point to a new mechanism of DDR activation through auto-/paracrine BE signaling of PLAUR/TLR4 membrane receptors (Figure 8). Our data suggest that a molecule serving as a DAMP ligand for TLR4/PLAUR receptor complex is exported from the nucleus of DNA-damaged cells and is released to the extracellular space. Thus, pharmacological blocking of nuclear export diminishes RAD51 activation, and conditioned media from DNA-damaged cells induced CHK1 activation in untreated cells in a PLAUR-dependent manner. The nature of TLR4/PLAUR ligand released by DNA-damaged cells is of great interest. Blocking antibody against HMGB1, a nuclear protein and known DAMP ligand of TLR4 did not affect CHK1 activation after Dox treatment. Another candidate is PLAU, the natural PLAUR ligand. As PLAU/PLAUR signaling affects MAPK pathway and generally pro-survival, it was reasonable to assume its role in DNA repair. However, blocking PLAU/PLAUR binding using antibody had also no effect on activation of CHK1, suggesting a different nature of ligand. TLR4 is strongly implicated in cancer pathology as a promoter of inflammation and angiogenesis^30^ and has implications in DNA repair.^31^ Our data suggest that TLR4-dependent DAMP signaling has a role in the activation of DNA repair pathways during rescue bystander signaling. Interestingly, our DNA repair assay in PLAUR and TLR4-expressing HEK-293 cells shows more-efficient functioning of only the HR pathway. Pharmacological inhibition of TLR4 prevented activation of CHK1, which confirms this mechanism. Indirect confirmation of our data comes from the report of CHK1 being a part of phospho-proteome of LPS-activated macrophages.^32^

It is of importance that PLAUR/TLR4 signaling targets CHK1, one of the most important DNA damage and repair regulators. Inhibiting CHK1 holds great promise to improve efficiency of DNA damaging drugs. Some tumors are also sensitive to CHK1 inhibitors alone.^33^ Several CHK1 inhibitors have been tested in phase I and II clinical trials. However, there are still inconsistencies regarding the efficiency of CHK1 inhibitors and optimal context for their applications such as for example in TP53 status. Earlier data suggested higher efficiency of CHK1 inhibitors in tumors with mutated TP53. Recent data on the contrary showed positive effects of CHK1 inhibition independently on TP53 status and thus challenged its predictive value. By comparing two cancer cell lines, MDA-MB-231 and HeLa cells, we observed that activation of CHK1 and DNA repair was affected similarly in both the cell lines. This corresponds to our previous data where we showed CHK1 activation to be PLAUR-dependent in vascular cells and in PLAUR-expressing HEK-293 cells. However, we observed that WT-TP53-expressing HeLa cells can sense the DNA damage and are capable of cell cycle arrest induction. On the contrary, TP53-deficient HeLa and MDA-MB-231 cells fail to arrest the cell cycle. The fate of cells differ accordingly; HeLa cells show increased cell death after DNA damage, whereas MDA-MB-231 cells continue to grow that results in accumulation of cells with nuclear abnormalities. The fate of MDA-MB-231 cells that harbor DNA damage, or use inefficient forms of DNA repair will be interesting to study. To exclude possible effects of HPV on TP53 function in HeLa cells, we used MCF-7 cells and obtained even more pronounced effects of PLAUR/TP53.

Our earlier observations show that PLAUR can regulate the ubiquitin-proteasome system and thus influence the DNA repair process.^34^ PLAUR/TLR4 signaling serves as an additional extracellular pathway of CHK1 activation. Therefore, PLAUR can strongly affect the process of DNA repair via several mechanisms. This is in agreement with extensive clinical data showing high PLAUR expression to be a prediction marker for poor survival and prognosis. Despite huge interest and promising results in animal models, therapeutic targeting of PLAUR has not been achieved yet. Reports suggest that non-proteolytic activity of PLAUR is more essential for cancer progression than promotion of cell invasion. Our data contribute to the understanding of the role of PLAUR in cancer and provide a rationale for addressing the correlation between PLAUR and TLR4 status, and DNA repair/CHK1 inhibitors efficiency. The data also allow speculations on the possibility of therapeutic targeting of PLAUR/TLR4 interaction. Such targeting may be essentially cancer cell-specific, as PLAUR expression in healthy tissues is low, and may also cause lesser toxic side-effects.

## Materials and Methods

### Cell culture

Cell lines HeLa, MDA-MB-231, MCF-7 and HEK-293 were purchased from ATCC. Cells were tested and authenticated by morphology and western blotting for specific markers in our laboratory. HeLa cells were cultured as recommended by the supplier in EMEM (Lonza, Basel, Switzerland) supplemented with 10% FBS, non-essential amino acids and penstrep (Millipore, Billerica, MA, USA). MDA-MB-231 and HEK-293 cells were cultured in DMEM (Lonza) supplemented with 10% FBS and penstrep. HEK-Blue hTLR cells were purchased from Invivogen (San Diego, CA, USA) and maintained as recommended by the supplier. Cell lines were tested for mycoplasma contamination every 6 months.

Cells were synchronized at G1/S-phase by double thymidine block as described before with minor modifications.^35^ In brief, thymidine (T1895, Sigma, St. Louis, MO, USA) was added to the culture at a final concentration of 2.5 mM. After 16 h, medium with excess thymidine was removed and cells were washed thrice with PBS. Fresh medium was added and after 8 h, the second round of thymidine (2.5 mM) was added and incubated for 16 h. Cells were released from the block and washed thrice with PBS, before treatment with Dox (Sigma D1515). Irradiation was performed using GammaCell 2000, having Cesium-137 as the radioactive source. Leptomycin-B (L2913) was from Sigma. CLI-095 was from Invivogen.

The following antibodies were used for western blotting: Tubulin (#2128), P-CHK1(345) (#2341), CHK1 (#2360), KU80 (#2753), TP53 (#2524) and V5-Tag (#13202) were from Cell Signaling Technology (Danvers, MA, USA); RAD51 (sc-8349) and GAPDH (sc-32233) were from Santa Cruz Biotechnology (Dallas, TX, USA); PLAUR (AF807), TLR1 (AF1475), TLR3 (AF1487), and TLR5 (MAB6704) from R&D Systems (Minneapolis, MN, USA). For immunocytochemistry *γ*-H2AX (#9718) was from Cell Signaling Technology; RAD51 (sc-8349) and TLR4 (H-80) from Santa Cruz biotechnology; P-RAD51(Tyr309) (ab111568) and P-RAD51(Thr315) (ab61111) were from Abcam (Cambridge, UK); PLAUR (3937) from Sekisui Diagnostics (Pfungstadt, Germany); neutralizing anti-HMGB1 (clone 3E8, 651402) were from Biolegend (San Diego, CA, USA); P-ERK antibody were from Cell Signaling Technology; PLAU/PLAUR binding was blocked by PLAUR (AF807) antibody from R&D Systems (San Diego, CA, USA).

### Transfection and viral infection

pDRGFP (Addgene, Cambridge, MA, USA, plasmid # 26475) and pCBASceI (Addgene plasmid # 26477) were a gift from Maria Jasin; EJ2GFP-puro (Addgene plasmid # 44025) was a gift from Jeremy Stark. pLenti6/V5-p53_wtp53 plasmid was a gift from Bernard Futscher (Addgene plasmid # 22945); pLKO-p53-shRNA-427 was a gift from Todd Waldman (Addgene plasmid # 25636); pLKO.1 puro was a gift from Bob Weinberg (Addgene plasmid # 8453). PLAURsi virus was used as previously described.^36^ FLAG-PLAUR vector was developed as follows. The QuikChange (Stratagene, San Diego, CA, USA) site-directed mutagenesis kit was used to make point mutations which introduced restriction nuclease *Ehe*I site in the structure of human PLAUR. Oligonucleotide primers containing the desired mutations were used to amplify a mutation-containing replica of the WT PLAUR in pBluescript SK+ (designated name pBS_uPAR) plasmid. EheI_dir: 5′-ACACTGCATGCACCGGGCGCCCCAAGAGGCTGG-3′ EheI_rev: 5′-CCAGCCTCTTGGGGCGCCCGGTGCATGCAGTGT-3′. In order to introduce FLAG epitope in the structure of PLAUR, pBS_uPAR was linearized with EheI and SphI restriction endonucleases and ligated with oligonucleotide duplexes: Primer uPAR_FLAG_dir: 5′-GACTACAAGGACGACGATGACAAGCTGCGGTGCATG-3′ Primer uPAR_FLAG_rev: 5′-CACCGCAGCTTGTCATCGTCGTCCTTGTAGTC-3′. This cloning step inserted DYKDDDDK peptide after PLAUR signal peptide. Lentivirus vector pWPTS-GFP (Tronolab, Lausanne, Switzerland) was modifiedby ligation of synthetic oligonucleotide duplex in *Sal*I and *Bam*HI restriction sites(5′-GATCCATATGCGGCCGCACTAGTTAATTAAG-3' 5′-TCGACTTAATTAACTAGTGCGGCCGCATATG-3′). Obtained vector was designated as pWPTS-Ad. Final lentivirus vector pWPTS-uPAR_FLAG was generated by digestion of pWPTS-Ad with *Sal*I and *Not*I and then ligation together with cDNA fragment of uPAR_FLAG.

Lentiviruses with VSV-G envelope were produced using HEK-293 cells, concentrated by ultracentrifugation and stored at −80 ºC. Viral titer was determined by LV Lentiviral Titer kit (MoBiTec, Göttingen, Germany) and viruses were used at a Multiplicity of infection (MOI) of 1–5 using polybrene (H9268, Sigma) at a concentration of 2 *μ*g/ml.

Scrambled control or PLAUR siRNA were obtained from Santa Cruz and transfected to MDA-MB-231 cells using Amaxa Nucleofector (Lonza) Program X-013. Mirus Transfection reagent was used for transfection of the cells. Media was changed after 24 h and cells were used for the experiments 48 h after transfection.

### DNA repair assays

HEK-293 cells and HEK-Blue hTLR were transfected with FLAG-PLAUR-expressing construct using Perfectin reagent. DNA repair assay was performed as previously described,^12^ HEK cells were transiently transfected with DRGFP (for HR) or EJGFP (for NHEJ) and pCBASceI plasmids using Perfectin transfection reagent. After 72 h, GFP-positive cells were counted by a FACS Canto flow cytometer (BD Biosciences, Franklin Lakes, NJ, USA), a minimum of 30 000 cells were acquired per sample. Comet assay was performed as described previously.^11^

### Western blotting and immunoprecipitation

Cells were lysed in RIPA buffer containing 1 mM PMSF, 1 mg/ml aprotinin, 1 mg/ml leupeptin, 1 mM Na3VO4, 1 mM NaF and incubated for 10 min at 4 °C. Cell lysates were subjected to sonication and then centrifuged at 10 000 rpm for 10 min. Typically, 50 *μ*g of protein was loaded on a polyacrylamide gel. PVDF membranes were blocked with 3% BSA, and probed with primary antibodies. Secondary antibodies conjugated to HRP were used to detect the proteins.

For co-immunoprecipitation cells were lysed by incubation on ice for 30 min, in a 20 mM Tris buffer (pH 7.4) containing 150 mM NaCl, 10 mM MgCl_2_, 2 mM EDTA, 10% Glycerol, 1% Triton X-100, 1 mM PMSF, 1 mg/ml aprotinin, 1 mg/ml leupeptin, 1 mM Na3VO4 and 1 mM NaF for 30 min on ice. The lysates were centrifuged at 10 000 rpm for 10 min. For immunoprecipitation 1000 *μ*g total cell lysate was incubated with 4 *μ*g of TLR4 (H-80) or PLAUR (C-16, Santa Cruz Biotechnology) antibody and Protein A/G PLUS agarose beads (Santa Cruz Biotechnology). Precipitates were washed three times in PBS buffer containing protease inhibitors and subjected to SDS- polyacrylamide gel electrophoresis.

### Immunofluorescence

Immunofluorescence was performed on cells grown overnight on coverslips and then treated with Dox or *γ*-Radiation; they were then fixed with 2% formaldehyde at the required time points, permeabilized with 0.1% Triton X-100 and blocked with 3% BSA in PBS at 4 °C overnight. Cells were labeled with primary and corresponding Alexa Fluor 488- or Alexa Fluor 594-conjugated secondary antibody (Invitrogen, Carlsbad, CA, USA) for 1 h at room temperature. DRAQ5 (Biostatus, Leicestershire, UK) was used for nuclear staining. Cells were then mounted with Aqua-Poly-Mount mounting medium (Polysciences, Warrington, PA, USA) and analyzed on a Leica TCS-SP2 AOBS confocal microscope. Images were quantified and analyzed by using ImageJ software.

Mean fluorescence intensity was calculated as previously described.^37^ Mean fluorescence intensity=Intensity of selection−(area of selection × background around the selection). Quantification of *γ*-H2AX foci in the nucleus was done by using the ImageJ macro PZ-FociEZ; which uses a DAPI image to define the nucleus and then *γ*-H2AX foci are counted within the region by using local maxima in fluorescence intensity, at least 100 nuclei were analyzed.^38, 39^

Duolink *In Situ* Proximity Ligation Assay (PLA) Probes (anti-rabbit PLA probe PLUS, anti-mouse PLA probe MINUS, and Duolink *In Situ* Detection Reagent Green) were purchased from Sigma-Aldrich and used according to the manufacturer's instructions. The number of PLA signals were quantified using ImageJ software.

### Cell cycle analysis

Cells were treated with 2* μ*M Dox for 1 h and then washed with PBS. Cells were detached with trypsin/EDTA at required time points and fixed with 70% ethanol and stored at −20 °C for at least 24 h. Cells were washed with PBS and stained with 300 *μ*l FxCycle PI/RNase Staining Solution (F10797, Thermofisher, Waltham, MA, USA) for 45 min. At least 30 000 cells were acquired using FACS Canto or LSRII flow cytometer (BD Biosciences, Franklin Lakes, NJ, USA). Data were analyzed using FlowJo software.

### Viability assay

Cell viability was assessed using CCK-8 kit (CK04, Dojindo, Kumamoto, Japan) as recommended; in brief cells were seeded at a density of 10 000 cells/well and incubated overnight. They were then treated with different concentrations of Dox for 1 h, washed with PBS and incubated. After 6 days of treatment 10 *μ*l of CCK-8 was added and incubated for 2 h, before reading the absorbance at 450 nm.

Colony-forming assay was performed by seeding Dox-treated cells at 3 000 cells per cell culture dish (ø10 cm) and culturing for 6 days. Cells were stained using Diff-Quik cell staining kit (Dade Behring, Deerfield, IL, USA). Dishes were photographed and colonies were counted using ImageJ software.

### Propidium iodide (PI) staining for dead cells

Cells in 6-cm dishes were treated overnight with 2 *μ*M Dox; they were then washed three times with PBS and incubated with fresh media. After 48 h, cells were trypsinised; there were also many floating cells, which were also collected. They were washed once with PBS, and suspended in PBS containing PI (0.5 mg/ml). Cells were immediately acquired using a LSRII flow cytometer (BD Biosciences). Data were analyzed using FlowJo software and the number of PI stained dead cells were plotted on the graph.

### Quantitative RT-PCR analysis

Total RNA was isolated from VSMC using RNeasy miniprep kit (Qiagen, Hilden, Germany). Real-time quantitative RT-PCR was performed on a LightCycler 480 Real-Time PCR System using LightCycler 480 RNA Master Hydrolysis probes (Roche Diagnostics, Basel, Switzerland). The oligonucleotide sequence of human uPA primers and probes were (sense 5'-ACTGCAGGAACCCAGACAACC-3' antisense 5′-TGGACAAGCGGCTTTAGGC-3′ probe 6-FAM-AGGCGACCCTGGTGCTATGTGCAG-TAMRA; GUSB was used as a housekeeping gene (sense 5′-GTGGTGCTGAGGATTGGCA-3′ antisence 5′-TAGCGTGTCGACCCCATTC-3′ Probe 6-FAM- TGCCCATTCCTATGCCATCGTGTG-TAMRA).

### Statistics

All experiments were repeated at least three times. Error bars are represented in terms of s.d. Graphpad prism software was used to perform an unpaired *t*-test assuming equal variance between the groups. *P*-values denoted are according to the following scale; ns (*P*>0.05), *(*P*≤0.05), **(*P*≤0.01), ***(*P*≤0.001), ****(*P*≤0.0001).

## Abbreviations

ATM: ataxia telangiectasia mutated
ATR: ataxia telangiectasia mutated and Rad3-related protein
BE: bystander effect
CHK1: checkpoint kinase 1
DSBs: double-strand breaks
DDR: DNA-damage response
HR: homologous recombination repair pathway
MAPKAPK: mitogen-activated protein kinase-activated protein kinase 2
NHEJ: non-homologous end joining
PKB/AKT: protein kinase B alpha
PLAUR: urokinase-type plasminogen activator receptor
PLAU: urokinase-type plasminogen activator
p90: transferrin receptor
RS: rescue signal
RAD51: RAD51 recombinase
RSK: ribosomal protein S6 kinase A1
TLR4: toll-like receptor 4
TP53: tumor protein P53
