# Supplementary material for: CHK1 and RAD51 activation after DNA damage is regulated via urokinase receptor/TLR4 signaling
Source: Cell Death Dis. 2016 Sep 29;7(9):e2383–. doi: 10.1038/cddis.2016.291 (PMC5059885; doi:10.1038/cddis.2016.291)
Supplement: Supplementary Figures [file cddis2016291x1.pdf]

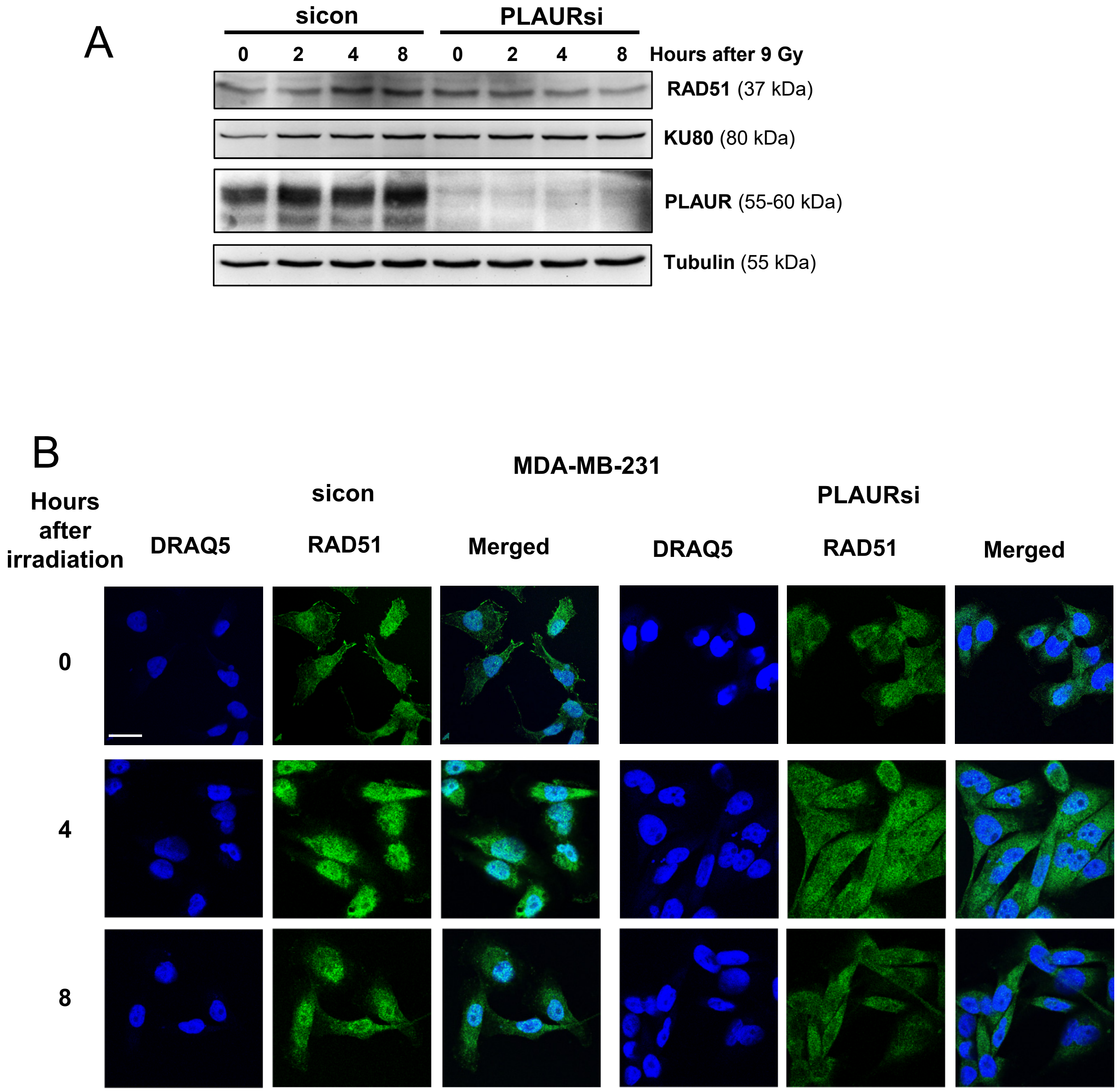

Supplementary Figure S1: A. sicon and PLAURsi, MDA-MB-231 cells were treated with radiation of 9 Gy and lysates were made at indicated time points. Western blotting was performed to check for the expression of RAD51 and KU80. **B.** MDA-MB-231 cells silenced for PLAUR were irradiated at 9 Gy, fixed after 4, 8 h and nuclear translocation and foci formation of RAD51 was detected by immunofluorescence (scale bar - 20 µm).

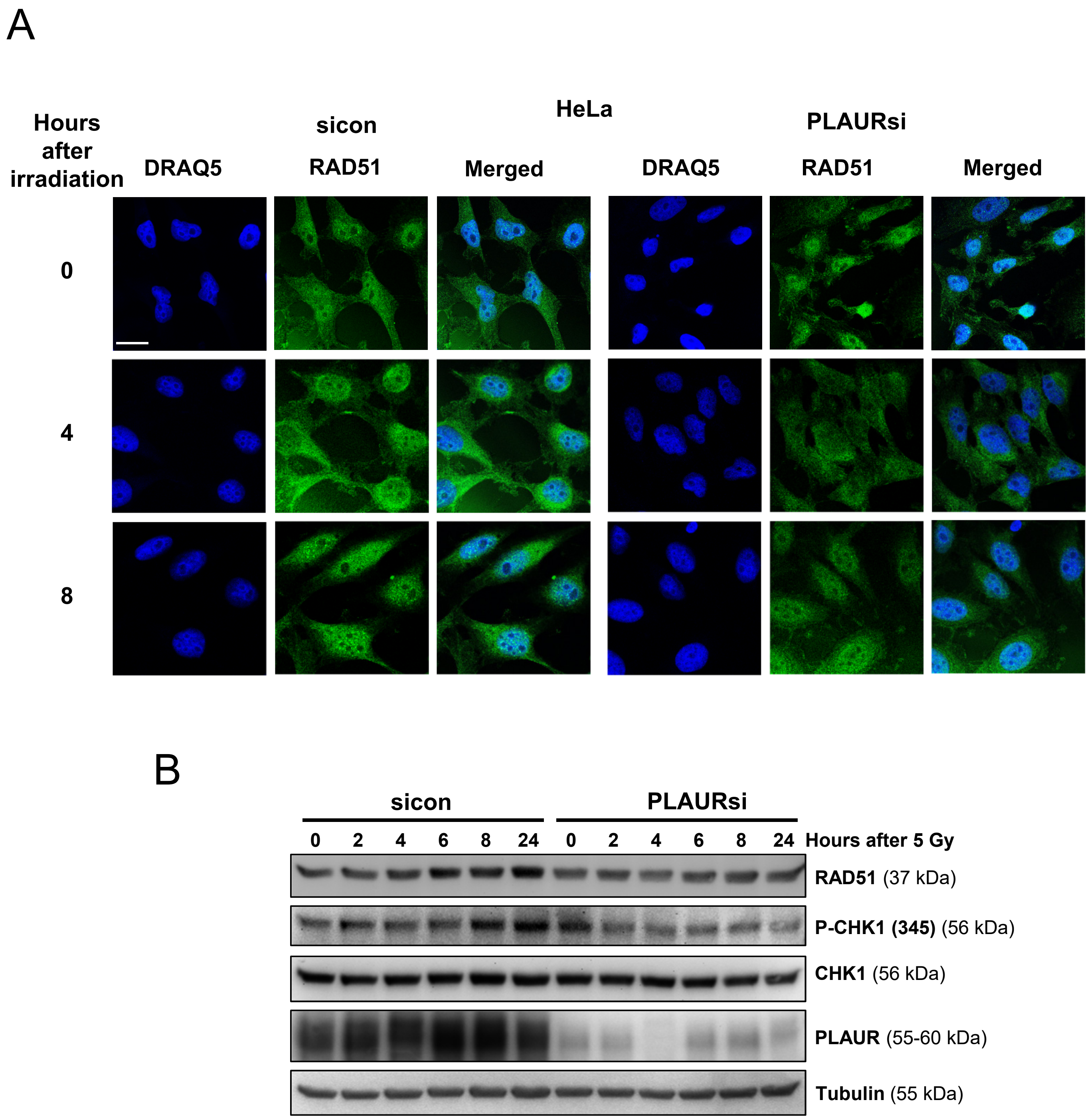

Supplementary Figure S2: A. HELA cells silenced for PLAUR were irradiated at 9 Gy, fixed after 4, 8 h and nuclear translocation and foci formation of RAD51 was detected by immunofluorescence (scale bar - 20 μm). **B.** MDA-MB-231 cells silenced for PLAUR were synchronized by double thymidine block and treated with radiation of 5 Gy, lysates were made at indicated time points. Western blotting was performed for the expression of RAD51, P-CHK1 and CHK1.

A

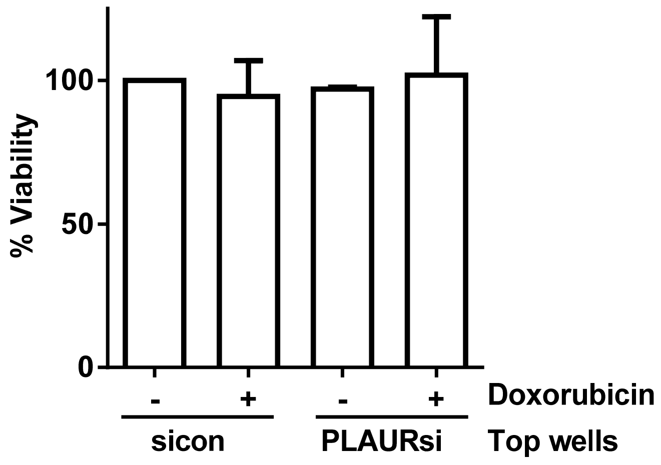

B

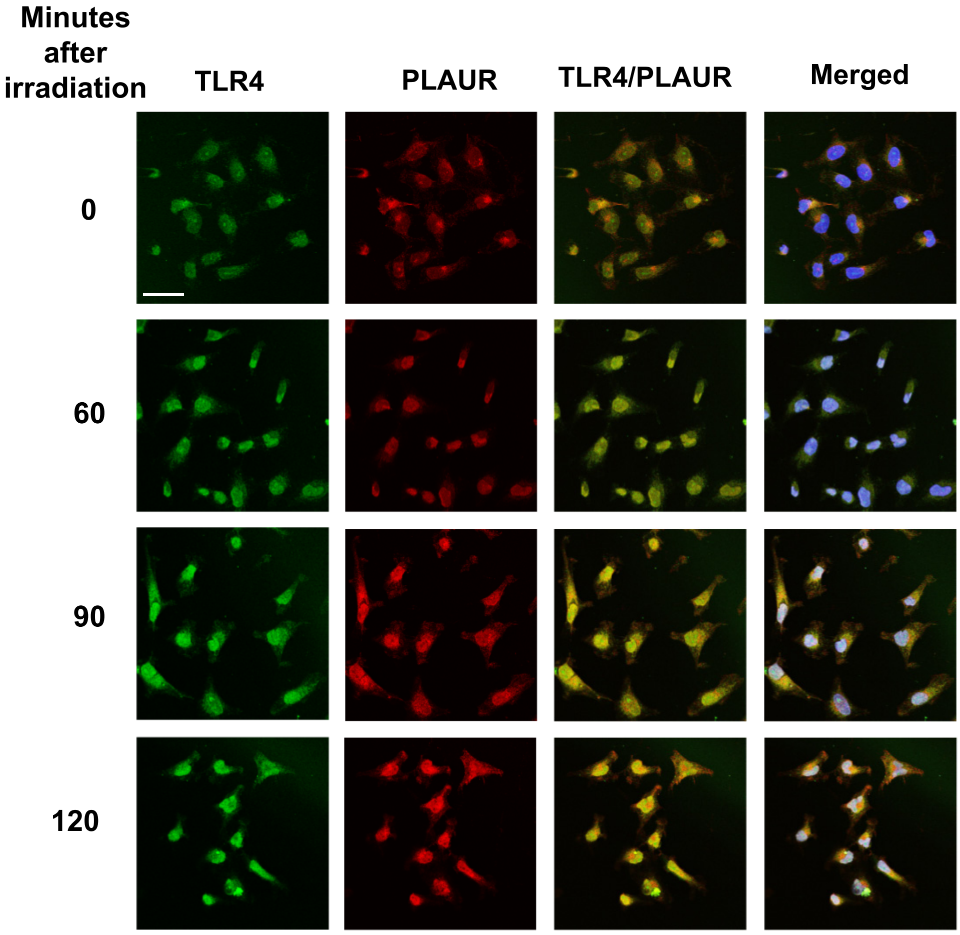

Supplementary Figure S3: A. sicon and PLAURsi HeLa cells were co-cultured using transwell plates, cells in the inserts were treated with doxorubicin (2 µM) for 1 h, and then placed in co-culture with untreated cells. Graph represents the viability of cells in the bottom well assessed after 6 days using CCK-8 (data are represented as mean +/- SD). **B.** HeLa cells irradiated at 9 Gy were fixed after 60, 90, 120 min and immunofluorescence was performed to detect co-localization of PLAUR and TLR4 (scale bar - 40 µm).

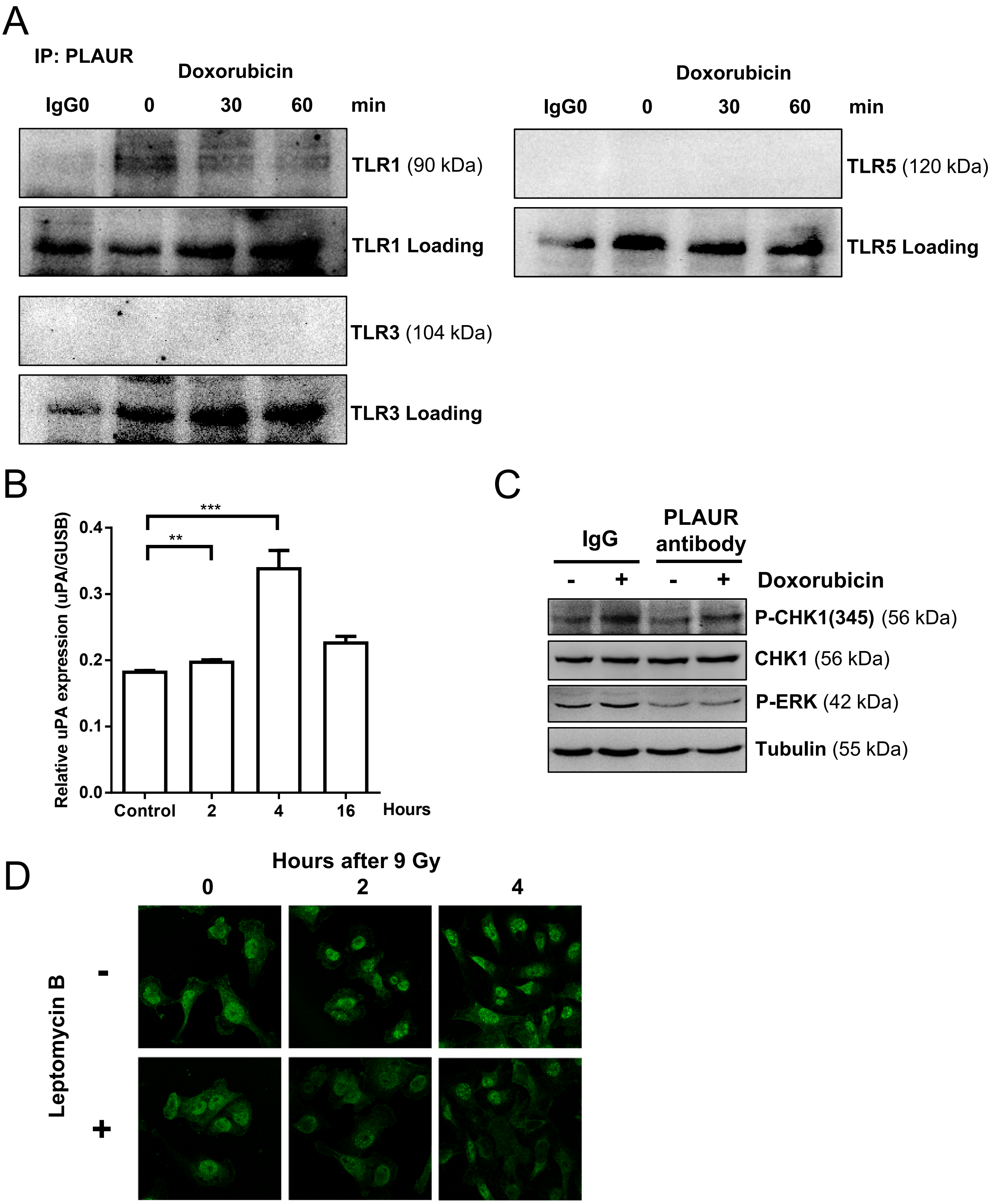

Supplementary Figure S4: A. Immunoprecipitation of PLAUR from Doxorubicin treated HeLa cells, followed by western blotting for detection of interacting TLR1, TLR3 and TLR5 proteins. **B.** Quantitative real time PCR for detection of mRNA levels of uPA in doxorubicin treated HeLa cells (data are represented as mean +/- SD). **C.** PLAUR blocking antibodies were used to inhibit binding of PLAU to PLAUR, cells were treated with Doxorubicin and western blotting was performed to detect P-CHK1 and P-ERK. **D.** MDA-MB-231 cells were pre-treated with Leptomycin-B to block nuclear export, DNA damage was then induced by radiation of 9 Gy. Cells were fixed and RAD51 was detected by immunofluorescence. Scale bar - 20 μm.

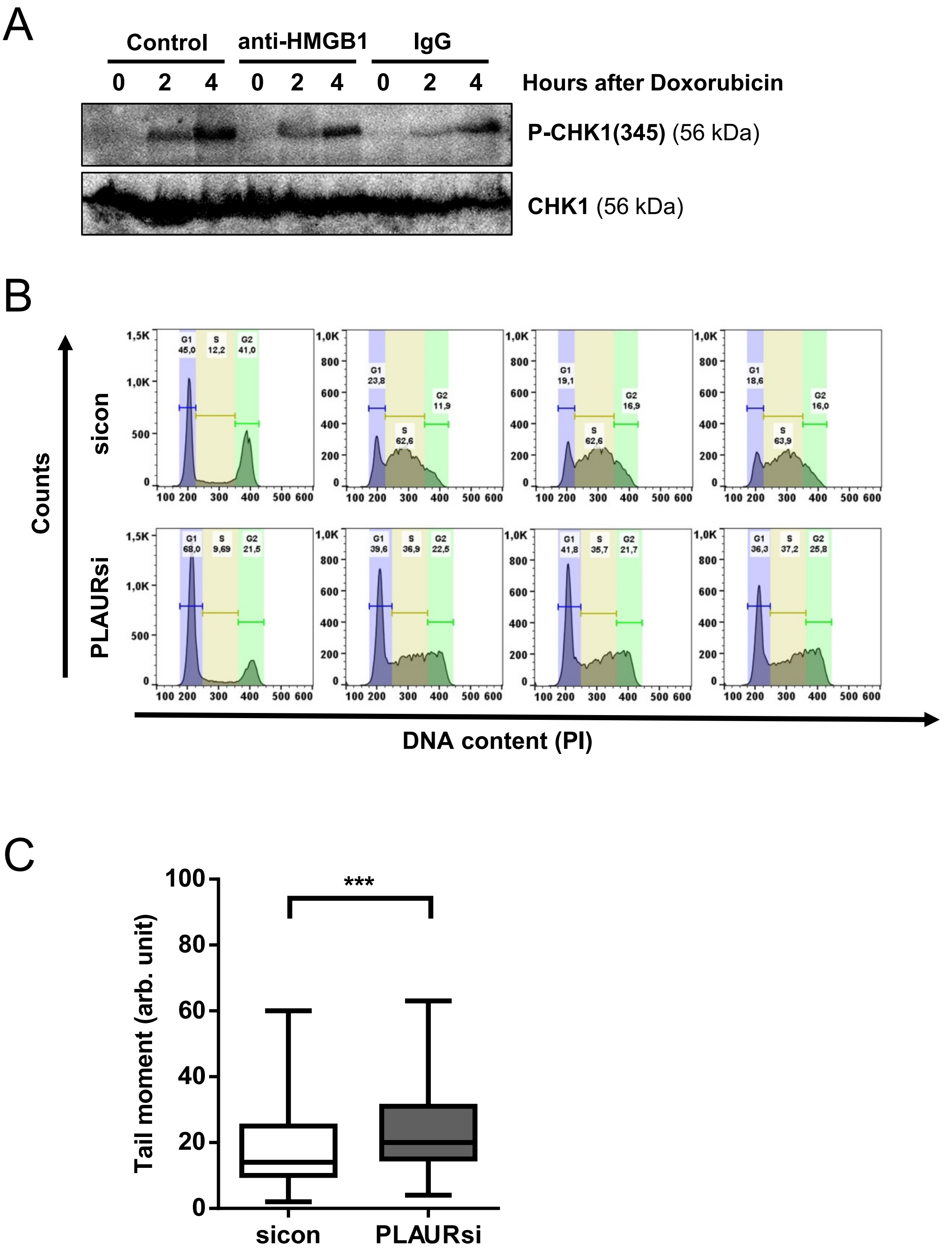

Supplementary Figure S5: A. Neutralizing HMGB1 antibodies were used to block its binding to TLR4, HeLa cells were treated with Doxorubicin as indicated and P-CHK1 was detected by western blotting **B.** Cell cycle analysis of MDA-MB-231 synchronized by double thymidine block and treated with doxorubicin (2 µM) for 1 h, cells were fixed at the indicated timepoints, stained with PI and analyzed by FACS. **C.** Comet assay indicating the DNA repair efficiency in sicon and PLAURsi cells treated with doxorubicin. (data are represented as mean +/- SD).

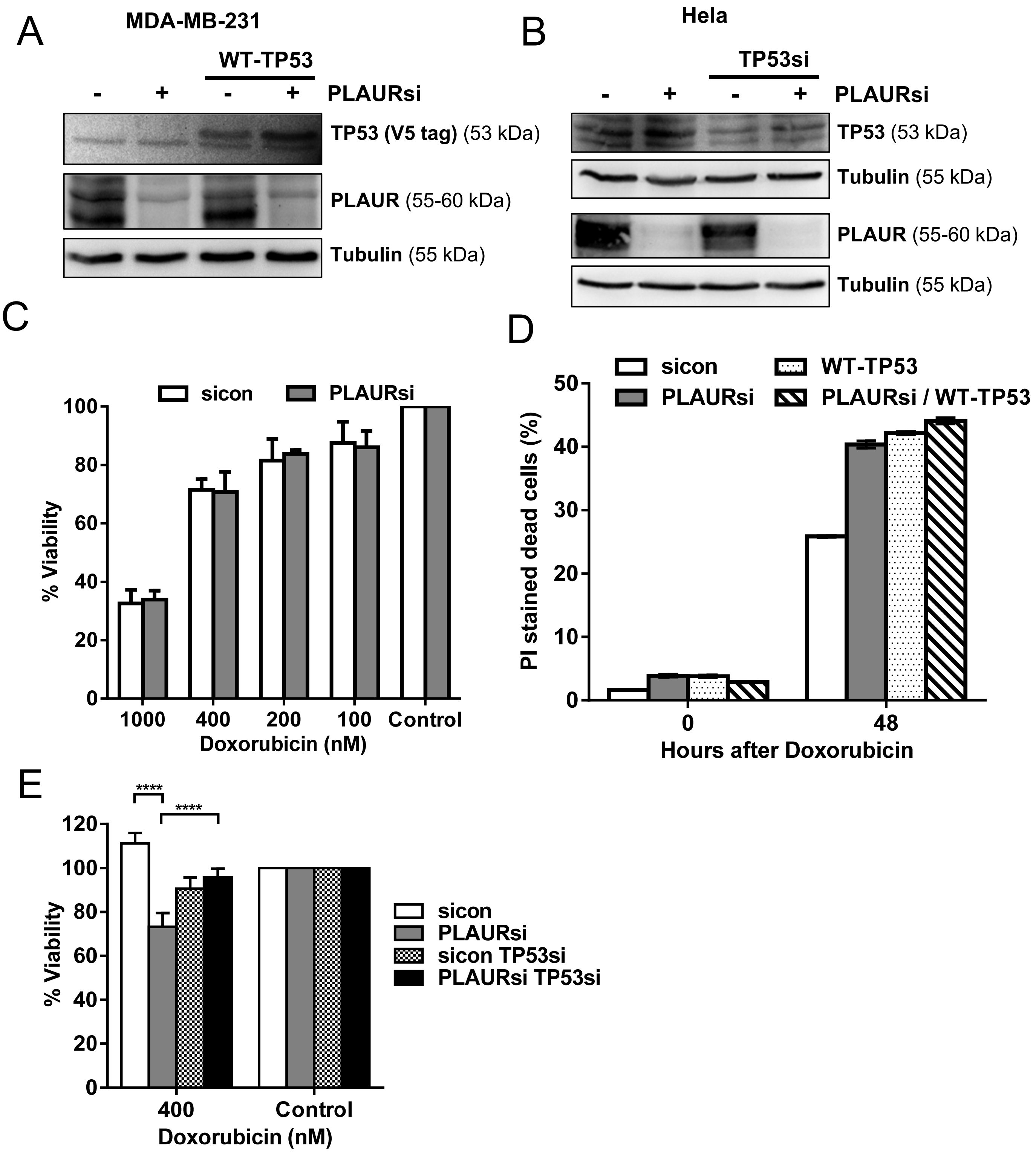

Supplementary Figure S6: A and **B.** Western blots showing expression of WT-TP53 in MDA-MB-231 (A) and silencing of TP53 in HeLa cells (B), lentiviral constructs were used to express or silence TP53. **C.** MDA-MB-231 silenced for PLAUR were treated with the indicated concentrations of doxorubicin for 1 h, cell viability was measured using CCK-8 kit 6 days after treatment. **D.** sicon and PLAURsi MDA-MB-231 cells were transduced with lentiviral constructs to express WT-TP53, and treated overnight with doxorubicin (2 μM), after 48 h cells were stained with PI and acquired by FACS to detect the number of dead cells. **E.** sicon and PLAURsi HeLa cells were transduced with lentiviral constructs to downregulate TP53, and then treated with 400 nM of doxorubicin for 1 h. Cell viability was measured using CCK-8 kit 6 days after treatment. All data are represented as mean +/- SD.
